# Expression of concern: Layer-by-layer aqueous synthesis, characterization and fluorescence properties of type-II CdTe/CdS core/shell quantum dots with near-infrared emission

**DOI:** 10.1039/d0ra90102d

**Published:** 2020-10-19

**Authors:** Laura Fisher

**Affiliations:** Royal Society of Chemistry Thomas Graham House, Science Park, Milton Road Cambridge UK CB4 0WF advances-rsc@rsc.org

## Abstract

Expression of concern for ‘Layer-by-layer aqueous synthesis, characterization and fluorescence properties of type-II CdTe/CdS core/shell quantum dots with near-infrared emission’ by Rijun Gui *et al.*, *RSC Adv.*, 2013, **3**, 20959–20969. DOI: 1039/C3RA43120G

RSC Advances is publishing this expression of concern in order to alert our readers that sections of the TEM images presented in Fig. 3a–f of this paper have been reproduced to represent different materials in at least eleven subsequent articles^1–11^ published by Rijun Gui *et al.*

The authors assert that the data presented in this RSC Advances article is correct and have provided raw data that matches Fig. 3a–f in the published article. However, they have not been able to provide a satisfactory explanation for all of the duplicated images that appear in the later publications. An expression of concern will continue to be associated with this article until we are able to obtain conclusive evidence about the reliability of this RSC Advances article.

Laura Fisher

Date: 21st September 2020

Executive Editor, RSC Advances

## Supplementary Material
